# JG26 attenuates ADAM17 metalloproteinase-mediated ACE2 receptor processing and SARS-CoV-2 infection in vitro

**DOI:** 10.1007/s43440-024-00650-0

**Published:** 2024-09-18

**Authors:** Valentina Gentili, Silvia Beltrami, Doretta Cuffaro, Giorgia Cianci, Gloria Maini, Roberta Rizzo, Marco Macchia, Armando Rossello, Daria Bortolotti, Elisa Nuti

**Affiliations:** 1 Department of Chemical, Pharmaceutical and Agricultural Sciences, University of Ferrara, Via Luigi Borsari 46, Ferrara, 44121 Italy; 2 Department of Pharmacy, University of Pisa, Via Bonanno 6, Pisa, 56126 Italy; 3 Clinical Research Center, LTTA, University of Ferrara, Ferrara, Italy

**Keywords:** ADAM17, SARS-CoV-2, Antiviral activity, Arylsulfonamido-based hydroxamic acid, sACE2

## Abstract

**Background:**

ADAM17 is a metalloprotease implicated in the proteolysis of angiotensin-converting enzyme 2 (ACE2), known to play a critical role in the entry and spread of SARS-CoV-2. In this context, ADAM17 results as a potential novel target for controlling SARS-CoV-2 infection.

**Methods:**

In this study, we investigated the impact on ACE2 surface expression and the antiviral efficacy against SARS-CoV-2 infection of the selective ADAM17 inhibitor JG26 and its dimeric (compound **1**) and glycoconjugate (compound **2**) derivatives using Calu-3 human lung cells.

**Results:**

None of the compounds exhibited cytotoxic effects on Calu-3 cells up to a concentration of 25 µM. Treatment with JG26 resulted in partial inhibition of both ACE2 receptor shedding and SARS-CoV-2 infection, followed by compound **1**.

**Conclusion:**

JG26, an ADAM17 inhibitor, demonstrated promising antiviral activity against SARS-CoV-2 infection, likely attributed to reduced sACE2 availability, thus limiting viral dissemination.

**Supplementary Information:**

The online version contains supplementary material available at 10.1007/s43440-024-00650-0.

## Introduction

ADAM17, also known as tumor necrosis factor-α‐converting enzyme (TACE) is a transmembrane zinc metalloproteinase belonging to the family of “A Disintegrin And Metalloproteinase” (ADAMs) [1]. It plays a crucial role in regulating various cellular processes by mediating the shedding of several membrane-bound proteins [2, 3]. Among ADAM17 substrates are included: cytokines, growth factors, adhesion molecules, and receptors involved in immune responses, cell signaling, and inflammation [4]. By cleaving these proteins from the cell surface, ADAM17 exerts significant influence over cell-cell communication, signal transduction, and the overall inflammatory milieu within tissues [5, 6]. Although ADAM17 is considered a hot target for the treatment of cancer and inflammatory diseases, no ADAM17 inhibitors have yet reached the market [7]. This is mostly due to the high homology among active site of ADAMs and the other members of the metzincin superfamily, MMPs and ADAMTSs [8], that makes it very challenging to obtain selective ligands devoid of off-target toxicity in vivo [9]. In the context of viral infections, ADAM17 has emerged as a pivotal player in the entry and propagation of certain viruses, including SARS-CoV-2 [10]. This virus, responsible for the COVID-19 pandemic, is a member of the coronavirus family, which includes other well-known viruses such as SARS-CoV and MERS-CoV. The structure of SARS-CoV-2 shares similarities with these other coronaviruses but also possesses unique features that contribute to its infectivity and pathogenicity. At its core, the viral RNA is encapsulated by the nucleocapsid (N) protein, forming a protective shell. Surrounding this RNA-protein complex is the viral envelope, a lipid bilayer membrane that serves as the outer shell of the virus. Embedded within this lipid bilayer are three key structural proteins: the spike (S) protein, the membrane (M) protein, and the envelope (E) protein. These proteins play crucial roles in the virus entry into host cells, replication, and assembly of new viral particles [11]. The spike (S) protein of SARS-CoV-2 interacts with host cell receptors such as angiotensin-converting enzyme 2 (ACE2) to enter target cells. ADAM17 is implicated in the proteolytic processing of ACE2, which is an important vascular regulatory component in the renin-angiotensin-system [12]. Recently, controversial opinions have been raised about the role of these proteases in SARS-CoV-2 infection. In fact, if to one side, ADAM17-mediated ACE2 shedding might decrease ACE2 availability on the cell surface, on the other hand the increase of soluble ACE2 (sACE2) released by host cells could improve viral spread in tissues with low or no ACE2 expression [13, 14].

In particular, Yeung et al. using an infection-permissive human kidney cell line, showed that sACE2 is responsible for an alternative mechanism of entrance of SARS-CoV-2 in tissues where ACE2 receptor is poorly expressed [15]. sACE2 can bind S protein of SARS-CoV-2 in the extracellular space and the resulting complex can enter host cells through receptor-mediated endocytosis, via the AT1 surface receptor or via the vasopressin receptor AVPR1B, if vasopressin participates in the complex formation. Thus, inhibition of ADAM17 could potentially disrupt this mechanism, thereby reducing the ability of SARS-CoV-2 to infect host cells. Moreover, ADAM17-mediated shedding of pro-inflammatory cytokines and other immune-modulatory molecules contributes to the pathogenesis of inflammatory diseases, including COVID-19 [16]. Excessive production and release of cytokines, often referred to as a cytokine storm, are characteristic of severe cases of COVID-19 and are associated with poor clinical outcomes [17]. Therefore, targeting ADAM17 presents a dual therapeutic opportunity in the context of COVID-19 which can help to modulate the inflammatory response and alleviate the cytokine storm associated with severe disease, but also to impede SARS-CoV-2 entry and propagation.

**Fig. 1:**
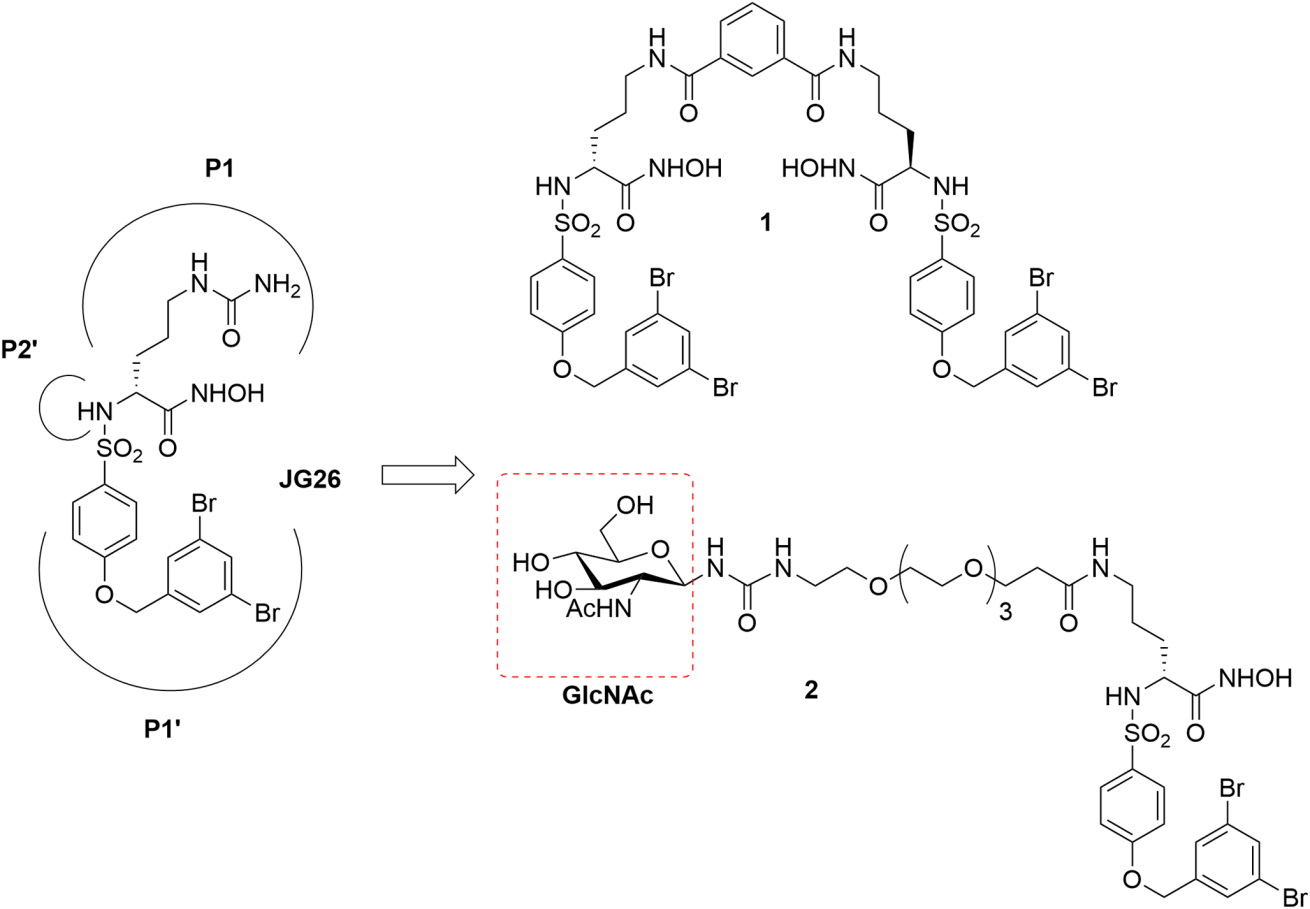
Chemical structures of ADAM17 inhibitors studied in the present work. **JG26** [18], compound previously published by our research group which served as a starting point for the design of compound **1** and compound **2**; GlcNAc (β-*N*-acetyl-D-glucosamine) moiety is highlighted in red

In the present work, we chose to study the selective ADAM17 inhibitor already reported by Rossello’s group, JG26 [18], and its derivatives compound **1** and compound **2** (Fig. 1). JG26 is an arylsulfonamido-based hydroxamic acid endowed with nanomolar activity against ADAM17 and able to block sALCAM release in ovarian cancer cell lines [18]. Compound **1** is a JG26 dimeric derivative developed as potential ADAM8 inhibitor which showed a stronger affinity for ADAM17 [19], while compound **2** is a JG26-glycoconjugate newly synthesized in this work. With the aim to optimize the water solubility and bioavailability of JG26 still maintaining its activity and selectivity for ADAM17, we adopted a previously reported [20] successful strategy based on the conjugation of a β-*N*-acetyl-d-glucosamine (GlcNAc) moiety (Fig. 1) with the ADAM17 inhibitor scaffold, using a 16 C-poly(ethylene glycol) (PEG) chain as a linker. GlcNAc is a component of glycoproteins, proteoglycans, glycosaminoglycans (GAGs) and other connective tissue building blocks naturally present in the human body [21]. In compound **2** the conjugation between GlcNAc and the ADAM17 inhibitor PEGylated chain was achieved through the introduction of a thioureido group, considered as a suitable linker biocompatible and stable in most bio-systems. All three compounds, presenting a common pharmacologically active scaffold but different structural modifications, polarity and surface area, were screened for their potential antiviral effects against SARS-CoV-2 infection, using an in vitro system based on Calu-3 human lung cells.

## Materials and methods

### Chemistry

^1^H and ^13^C NMR spectra were recorded on a Bruker Avance III HD 400 MHz spectrometer. Chemical shifts (δ) are reported in parts per million and coupling constants (*J*) are reported in hertz (Hz). ^13^C NMR spectra were fully decoupled. The following abbreviations were used to explain multiplicities: singlet (s), doublet (d), triplet (t), double doublet (dd), broad singlet (bs), and multiplet (m). Chromatographic separations were performed on silica gel columns by flash column chromatography (Kieselgel 40, 0.040–0.063 mm, Merck) or using ISOLUTE Flash Si II cartridges (Biotage). Reactions were followed by thin-layer chromatography (TLC) on Merck aluminum silica gel (60 F254) sheets that were visualized under an UV lamp and hydroxamic acids were visualized with FeCl_3_ aqueous solution. Evaporation was performed *in vacuo* by rotary evaporator. Sodium sulfate was always used as the drying agent. Commercially available chemicals were purchased from Sigma-Aldrich (Merck). Elemental analysis was used to determine the purity of target compounds. Analytical results are within ± 0.4% of the theoretical values. The ESI-MS spectra were recorded by direct injection at 5 (positive) and 7 (negative) µl min − 1 flow rate in an Orbitrap high-resolution mass spectrometer (Thermo, San Jose, CA, USA), equipped with HESI source.

### Synthesis of (4*R*)-4-(4-((3, 5-dibromobenzyl)oxy) phenylsulfonamido)-5-oxo-5-(((tetrahydro-2 H-pyran-2-yl)oxy)amino)pentan-1-aminium trifluoroacetate (4)

To a solution of compound **3** (533 mg, 0.7248 mmol) in dry CH_2_Cl_2_ (2.2 mL), cooled at 0 °C, trifluoroacetic acid (1.11 mL, 14.50 mmol) was added dropwise. The reaction was stirred at 0 °C for 30 min and at room temperature for 50 min, under argon atmosphere, then it was concentrated *in vacuo*. The crude product was washed three times with CH_2_Cl_2_ and concentrated *in vacuo*. It was then purified by flash chromatography using an ISOLUTE Si II 10 g cartridge (CHCl_3_/CH_2_Cl_2_ 20:1) to afford compound **4** as a white solid (202 mg, 37%). ^1^H NMR (400 MHz, DMSO-*d*_6_) δ: 1.44–1.59 (m, 4H), 2.66-2-73 (m, 2H), 3.59–3.63 (m, 1H), 5.21 (s, 2H), 7.16 (d, *J* = 8.9 Hz, 2H), 7.71–7.73 (m, 4H), 7.85 (bs, 3H), 7.94 (d, *J* = 8.8 Hz, 1H), 8.85 (bs, 1H), 10.65 (s, 1H).

### Synthesis of *tert*-butyl ((20*R*)-20-(4-((3, 5-dibromobenzyl)oxy)phenylsulfonamido)-15, 21-dioxo-21-(((tetrahydro-2 H-pyran-2-yl)oxy)amino)-3, 6, 9, 12-tetraoxa-16-azahenicosyl)carbamate (6)

To a solution of NHS-PEG derivative **5** [22] in DMF dry (2 mL), DIPEA dry (94 µL, 0.538 mmol) was added; then a solution of compound **4** in DMF (2 mL) was added dropwise. The final solution was stirred at room temperature overnight under argon atmosphere. The reaction was diluted with EtOAc, washed with HCl 1 N and brine. The combined organic layers were dried over Na_2_SO_4_ and concentrated *in vacuo*. The crude product was purified by flash chromatography using an ISOLUTE Si II (5 g) cartridge (100% CHCl_3_) to afford compound **6** as a brown oil (180 mg, 68%). ^1^H NMR (400 MHz, CDCl_3_) δ: 1.44 (s, 9H), 1.54–1.80 (m, 10H), 2.48–2.58 (m, 2H), 3.29–3.32 (m, 2H), 3.54–3.55 (m, 2H), 3.62–3.65 (m, 12H), 3.71–3.74 (m, 3H), 3.87–3.95 (m, 1H), 4.08–4.17 (m, 1H), 4.55–4.57 (m, 1H), 4.78 (bs, 1H), 5.03 (s, 2H), 5.06–5.15 (m,1H), 6.97–7.03 (m, 2H), 7.49–7.52 (m, 2H), 7.65 (s, 1H), 7.78–7.82 (m, 2H), 9. 95 (bs, 1H).

### (20*R*)-20-(4-((3, 5-dibromobenzyl)oxy)phenylsulfonamido)-15, 21-dioxo-21-(((tetrahydro-2 H-pyran-2-yl)oxy)amino)-3, 6, 9, 12-tetraoxa-16-azahenicosan-1-aminium trifluoroacetate (8)

To a solution of compound **6** (180 mg, 0.1821 mmol) in CH_2_Cl_2_ dry (0.6 mL), trifluoroacetic acid (0.279 mL, 3.643 mmol) was added dropwise at 0 °C. The reaction was maintained at 0 °C for 30 min and at room temperature for 30 min under argon atmosphere, then was evaporated. The crude product was co-evaporated with CH_2_Cl_2_ (3x) and then purified by flash chromatography using an ISOLUTE Si II cartridge (10 g) (CHCl_3_/CH_2_Cl_2_ 100:1) to afford the trifluoroacetate salt **8** as a white solid (75 mg; 41% yield). ^1^H NMR (400 MHz, DMSO-*d*_6_) δ: 1.14–1.80 (m, 10H), 2.26–2.32 (m, 2H), 2.88–2.99 (m, 4H), 3.48–3.69 (m, 15H), 3.80–3.86 (m, 2H), 4.30–4.35 (m, 1H), 4.96–4.99 (m, 1H), 5.18–5.19 (m, 1H), 5.22 (s, 2H), 7.08–7.16 (m, 2H), 7.64–7.87 (m, 9H), 9.75 (s, 1H).

### (2*R*, 3 *S*, 4*R*, 5*R*, 6*R*)-5-acetamido-2-(acetoxymethyl)-6-(3-((20*R*)-20-(4-((3, 5-dibromobenzyl)oxy)phenylsulfonamido)-15, 21-dioxo-21-(((tetrahydro-2 H-pyran-2-yl)oxy)amino)-3, 6, 9, 12-tetraoxa-16-azahenicosyl)thioureido)tetrahydro-2 H-pyran-3, 4-diyl diacetate (9)

To a solution of compound **8** (75 mg, 0.07529 mmol) and Et_3_N (11 µL, 0.07529 mmol) in CH_2_Cl_2_ (5 mL), a solution of commercial isothiocyanate β-*N*-acetyl-d-glucosamine **7** [20] (32 mg, 0.082 mmol) in CH_2_Cl_2_ (2 mL) was added dropwise. The reaction was stirred overnight at room temperature and evaporated *in vacuo*. The crude product was purified by flash chromatography using an ISOLUTE Si II (5 g) cartridge (CHCl_3_/CH_2_Cl_2_ 50:1), to afford the compound as a white solid (61 mg, 63% yield). ^1^H NMR (400 MHz, DMSO-*d*_6_) δ: 1.29–1.61 (m, 10H), 1.77 (s, 3H), 1.91 (s, 3H), 1.96 (s, 3H), 1.99 (s, 3H), 2.25–2.28 (m, 2H), 2.90–3.05 (m, 2H), 3.47–3.57 (m, 19H), 3.73–3.99 (m, 4H), 4.02–4.06 (m, 1H), 4.82 (t, *J* = 9.6 Hz, 1H), 4.95–5.11 (m, 1H), 5.18 (s, 2H), 5.55 (bs, 1H), 7.09–7.15 (m, 2H), 7.64–7.69 (m, 4H), 7.78–7.80 (m, 1H), 7.86–7.89 (m, 1H), 7.93–7.97 (m, 1H), 8.09 (d, *J* = 8.4 Hz, 1H), 9.72 (s, 1H).

### 1-(((2*R*, 3*R*, 4*R*, 5 *S*, 6*R*)-3-acetamido-4, 5-dihydroxy-6-(hydroxymethyl)tetrahydro-2 H-pyran-2-yl)amino)-*N*-((*R*)-4-(4-((3, 5-dibromobenzyl)oxy)phenylsulfonamido)-5-(hydroxyamino)-5-oxopentyl)-1-thioxo-5, 8, 11, 14-tetraoxa-2-azaheptadecan-17-amide (2)

To a solution of compound **9** (33 mg, 0.02596 mmol) in 500 µL of MeOH, NH_3_-MeOH 7 N (250 µL) was added. The reaction was stirred overnight at room temperature and finally evaporated *in vacuo* to afford the crude product as a yellow solid that was used in the next step without any further purification. To a solution of the crude in CH_2_Cl_2_ (350 µL), cooled at 0 °C, TFA was added dropwise (526 µL); the reaction was stirred at room temperature for 8 h and evaporated *in vacuo*. The crude product was triturated with Et_2_O (2x) and then with *n*-hexane (2x) to afford compound **2** as a colorless semisolid (23 mg, 85% yield). ^1^H NMR (400 MHz, DMSO-*d*_6,_) δ: 1.41–1.45 (m, 4H), 1.82 (s, 3H), 2.25–2.27 (m, 2H), 2.52–2.55 (m, 2H), 2.87–2.93 (m, 2H), 3.14–3.17 (m, 1H), 3.38–3.68 (m, 21H), 3.76–3.79 (m, 1H), 4.48–4.50 (m, 1H), 5.19 (s, 2H), 7.66–7.71 (m, 4H), 7.79–7.84 (m, 2H), 7.88–7.90 (m, 1H), 7.91-8.00 (m, 1H), 8.10–8.25 (m, 1H), 8.84 (m, 1H), 10.54 (s, 1H). ^13^C NMR (100 MHz, CD_3_OD) δ: 181.7, 174.8, 173.0, 160.4, 146.2, 138.4, 137.3, 134.5, 133.1, 129.6-127.8, 116.5, 84.5, 78.9, 75.8, 71.8, 70.2, 68.7, 62.6, 61.9, 56.1, 53.0, 26.15; 30.25. HRMS (ESI, *m/z*) calculated for C_38_H_56_N_6_O_15_S_2_Br_2_ [M − H]−: 1057.15390; found: 1057.15503; [M + Cl]−: 1093.13058; found: 1093.13196. Elemental analysis calcd (%) for C_38_H_56_N_6_O_15_S_2_Br_2_ : C 43.02, H 5.32, N 7.92; found: C 43.22, H 5.41, N 8.00.

### Biological evaluation

#### Enzyme inhibition assays

Recombinant human MMP-14 catalytic domain was a kind gift of Prof. Gillian Murphy (Department of Oncology, University of Cambridge, UK). Pro-MMP-1 (444208), pro-MMP-2 (PF037), pro-MMP-9 (PF038) and recombinant human ADAM17 (PF133) were purchased from Merck Millipore (Burlington, MA, USA). Recombinant human ADAM10 (936-AD) was purchased from R&D Systems (Milan, Italy). *p*-Aminophenylmercuric acetate (APMA, A9563) was from Sigma-Aldrich (Milan, Italy).

Proenzymes were activated immediately prior to use with APMA 2 mM for 1 h at 37 °C for MMP-2, APMA 2 mM for 2 h at 37 °C for MMP-1 and APMA 1 mM for 1 h at 37 °C for MMP-9.

For assay measurements, the compound stock solution (10 mM in DMSO) was further diluted for each MMP in the fluorometric assay buffer (FAB: Tris 50 mM, pH = 7.5, NaCl 150 mM, CaCl_2_ 10 mM, Brij 35 0.05% and DMSO 1%) following the protocol already reported [18]. Activated enzyme (final concentration 0.56 nM for MMP-2, 1.3 nM for MMP-9, 1.0 nM for MMP-14 cd, 5.0 nM for ADAM17, 20 nM for ADAM10 and 2.0 nM for MMP-1) and inhibitor solutions were incubated in the assay buffer for 3 h at 25 °C. ADAM17 was incubated for 30 min at 37 °C and ADAM10 for 1 h at 37 °C in a different buffer at pH 9 (Tris 25 mM, ZnCl2 25 µM, Brij-35 0.005%). After the addition of 200 µM solution of the fluorogenic substrate Mca-Lys-Pro-Leu-Gly-Leu-Dap(Dnp)-Ala-Arg-NH_2_ (444282, Merck Millipore) in DMSO (final concentration 2 µM for all enzymes and 10 µM for ADAM10), the hydrolysis was monitored every 15 s for 15 min recording the increase in fluorescence (λex = 325 nm, λem = 400 nm) using a SpectraMax Gemini XPS (Molecular Devices, Sunnyvale, CA) plate reader. The assays were performed in triplicate in a total volume of 200 µL per well in 96-well microtitre plates (Corning, black, NBS). The MMP inhibition activity was expressed in relative fluorescence units (RFU). Percent of inhibition was calculated from control reactions without the inhibitor. IC_50_ was determined using the formula: *V*_i_/*V*_o_ = 1/(1 + [I]/ IC_50_), where *V*_i_ is the initial velocity of substrate cleavage in the presence of the inhibitor at concentration [I] and *V*_o_ is the initial velocity in the absence of the inhibitor. Results were analyzed using SoftMax Pro software version 5.4.3 and Origin 6.0 software.

### Cell culture and treatments

Calu-3 (ATCC HTB-55) human epithelial lung cells and VERO C1008 (Vero E6, ATCC CRL1586TM) African green monkey epithelial kidney cells were cultured in Eagle’s minimal essential medium (MEM) with nonessential amino acids (Lonza Biosciences), containing 10% heat-inactivated fetal calf serum (FCS, Gibco), penicillin–streptomycin solution (1X, Gibco), and L-glutamine (2 mM; Lonza Biosciences), at 37 °C with a 90–95% of relative humidity and 5% of CO_2_ concentration.

Cells were exposed to JG26, compound **1** and compound **2** at the concentration of 5, 7.5, 10, 15, 20 and 25 µM to perform viability and antiviral activity evaluations.

### MTT assay for cell viability

Cell viability was spectrophotometrically assessed using the MTT (3-(4,5-dimethilthiazol-2yl)-2,5-diphenyl tetrazolium bromide) assay (Roche Diagnostics Corporation, Indianapolis, IN, USA) with absorbance measurements taken at 570 nm in accordance with the manufacturer’s guidelines. Cells were exposed to serial dilutions of compounds for 24 and 48 h and to DMSO 25 µM and Phorbol 12-myristate 13-acetate (PMA) 2 µM [23] as negative and positive controls, respectively. The experiments were performed in triplicate and data reported as mean % ± SD compared to untreated samples (100% viability). The highest concentration associated to cell viability higher than 80% was selected for further experiments.

### SARS-CoV-2 propagation and infection

A SARS-CoV-2 inoculum was obtained from a nasopharyngeal swab of a COVID-19 patient (Caucasian male of Italian descent; genome sequences registered in GenBank (SARS-CoV-2-UNIBS-AP66: ERR4145453; this SARS-CoV-2 isolate clustered in the B1 clade). The virus was kindly provided by Professor Arnaldo Caruso (University of Brescia, Italy). SARS-CoV-2 was cultured in Vero E6 cells and quantified using a plaque assay titration method as described elsewhere [24]. Calu-3 cells were then infected with a multiplicity of infection (MOI) of 0.1 for 2 h at 37 °C, as outlined in earlier reports [25]. The supernatants from the infected cells were collected after 48 h for further analysis. All experiments were conducted under biosafety level-3 (BSL-3) conditions.

### Antiviral activity evaluation

To perform antiviral assay, Calu-3 cells were seeded in 24-well plates, infected with SARS-CoV-2 and treated with compounds at the highest concentration tested during MTT, resulted not cytotoxic. The treatments were performed after the virus-absorption step. After 24 and 48 h of infection, RNA was isolated from 500 µL of cell culture supernatants following centrifuge-clarification (16,000 g × 10 min) using a PureLink Viral RNA/DNA Mini Kit (Thermo Fisher, Milan, Italy) according to the manufacturer’s instructions. RNA was recovered with 15 µL of RNase-free water and kept at − 80 °C until use. Reverse transcriptase SuperScript IV VILO (Thermo Fisher, Milan, Italy) was used to convert extracted RNA in cDNA. The quantification of SARS-CoV-2 genomes was obtained by amplification of the S gene using a PowerUp SYBR Green Master Mix (Thermo Fisher, Milan, Italy) with the following primers: RBD-qF1:5′-AATGGTTTAACAGGCACAGG-3′ and RBD-qR1:5′-CTCAAGTGTCTGTGGATCACG-3′. A synthetic sequence of dsDNA (gBlock, Integrated DNA Technologies, Coralville, IA, USA) containing the RBD sequence was serially diluted to create a standard curve ranging from 10^8^ to 10^2^ copies, allowing absolute quantification.

In order to assess the levels of infective SARS-CoV-2 released by treated or untreated cells, a plaque assay was performed. Vero E6 were seeded in 12-well plates and infected with serial dilution (10-fold) of the supernatants derived from treated and infected Calu-3 cells. Plaques were visible five days after infection, then cells were methanol-fixed, stained with crystal violet (0.1%) and plaques were manually counted [26] (microscope NeXcope NE620). The experiments were run in triplicate.

### Immunofluorescence assay

Calu-3 cells were incubated with a specific antibody against human angiotensin-converting enzyme 2 (ACE2) (SN0754 Thermo Fisher; Italy) as previously reported [27, 28], followed by incubation with the FITC goat antirabbit IgG (H + L) secondary antibody (Thermo Fisher, Milan, Italy). Immunofluorescence was observed by fluorescence microscopy (Nikon Eclipse TE2000S, Milan, Italy). DNA was stained using DAPI (Thermo Fisher, Milan, Italy) to identify cell nuclei and reported as relative fluorescence units (RFU) compared to untreated control sample, by immunofluorescence quantification derived by the analysis of 5 different images derived by biological replicates, using Qupath software. In Brief, for each condition, the percentage of ACE2-positive stained cells was obtained considering the total DAPI-stained nuclei as 100% and then normalized on untreated control sample result and reported as fold difference value.

### Plaques analysis

After crystal violet (0.1%) staining for antiviral activity assessment, plaques forming unit were identified and measured in size. Dimension of plaques derived by cytopathic effect of SARS-CoV-2 in Vero E6 cells was evaluated by microscopy (NeXcope NE620). For each well, 5 different fields were manually selected, and at least 10 plaques for each condition were identified and measured using NeXcope visualization software (Capture V.2.0) and reported as mean diameter (mm) ± SD.

### Statistical analysis

All the statistical analysis were performed using GraphPad Prism version 10 software (GraphPad, La Jolla, CA, USA). Each experiment was performed in triplicate and results are reported as mean ± standard deviation (SD). Immunofluorescence staining was analyzed with QuPath software and reported as relative fluorescence units (RFU).

Data distribution normality was defined using the Kolmogorov-Smirnov test. Variance analysis was performed using one-way ANOVA or two-way ANOVA, in case of independent variables, respectively, and reported as F_DFn, DFd_ (DFn = k-1, degrees of freedom; DFd = error degrees of freedom, N-k, where N is the total number of observations and k is the number of groups). Significant ANOVA results were followed by Dunnett’s or Dunnett’s T3 multiple comparison post hoc test and p values were reported on graphs. P values < 0.05 were considered significantly different.

For each compound, half maximal inhibitory concentration (IC_50_), concentration of cytotoxicity 50% (CC_50_), half maximal effective concentration (EC_50_) were calculated by interpolation using GraphPad software and Selectivity Index (SI) was obtained as CC_50_/EC_50_ ratio.

## Results

### Chemistry

**Fig. 2:**
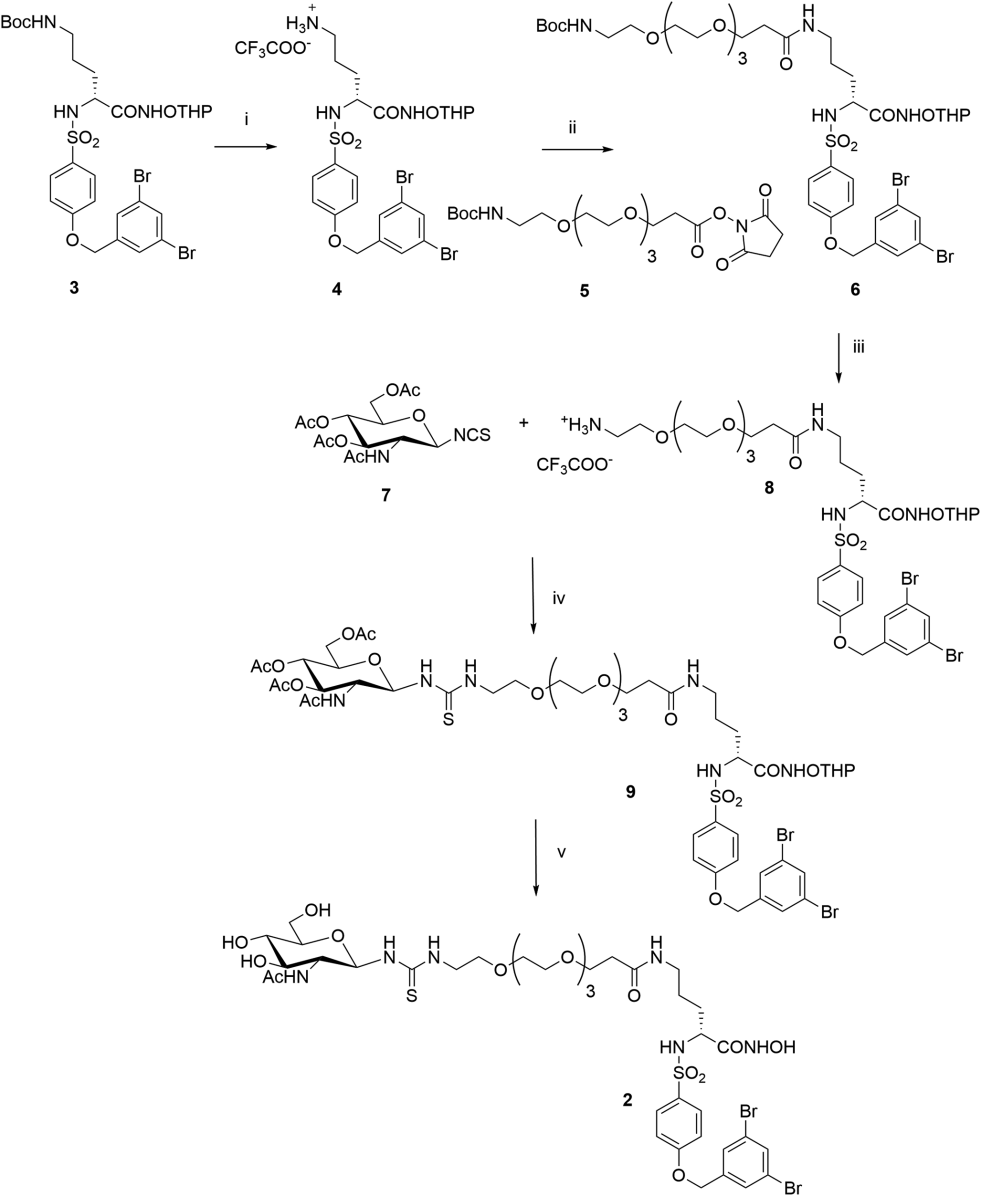
Synthesis of compound **2**. Reagents and conditions: **i**) TFA, dry DCM, 0 °C, 1 h, 37%; **ii**) **5**, dry DIPEA, dry DMF, 18 h, rt, 68%; **iii**) TFA, dry DCM, 0 °C, 1 h, 41%; **iv**) **7**, Et_3_N, dry DCM, 18 h, rt, 63%; **v**) (1) NH_3_-MeOH 7 N, MeOH, 18 h, rt; (2) TFA, dry DCM, 8 h, rt (85% over two steps)

JG26 and compound **1** were prepared as previously reported [18, 19]. The synthetic pathway followed to obtain compound **2** is described in Fig. 2.

The previously described NH-Boc and THP-protected hydroxamate **3** [22] was converted into the protected hydroxamate **4** as trifluoroacetate salt by selective hydrolysis with trifluoroacetic acid (TFA) in dichloromethane (DCM) under controlled conditions (1 h, 0 °C). The coupling between the amino group of compound **4** and the NHS-ester activated spacer **5** [22] afforded the PEGylated intermediate **6**. Again, a selective hydrolysis of NH-Boc group on compound **6** performed under controlled conditions afforded the amine **8** as trifluoroacetate salt. The condensation between amine **8** and the already known β-*N*-glycosyl isothiocyanate **7** [20], gave the peracetyl glycoconjugate **9**, which after treatment with methanolic ammonia 7 N followed by acid hydrolysis with TFA afforded the final glycoconjugate hydroxamate **2** in good yields.

### Biological evaluation

First, the newly synthesized compound **2** was tested on human recombinant ADAM17 by a fluorometric assay, which uses a fluorogenic peptide [29] as the substrate, to verify its affinity for the target enzyme. Inhibition against MMP-1, -2, -9, -14 and ADAM10 was also tested to evaluate the selectivity over other metalloproteases belonging to the same superfamily. The new results are reported in Table 1 in comparison with activity data already determined for JG26 [18] and compound **1** [19].

**Table 1:** In vitro inhibitory activity (IC_50_, nM) of compound JG26 and its derivatives (compound **1**, compound **2**) evaluated on isolated enzymes by a fluorometric assay

Compound	ADAM17	ADAM10	MMP-1	MMP-2	MMP-9	MMP-14
**JG26** [18]	1.9	150	> 500,000	240	1630	19,500
Compound **1** [19]	24	10,000	> 200,000	3000	8800	50,000
Compound **2**	5.5	810	> 200,000	320	1771	70,700

Reported data are the average of three determinations with a SD of < 10%. IC_50_, half maximal inhibitory concentration; MMP, matrix metalloproteinase; ADAM, a disintegrin and metalloproteinase

Enzymatic results showed that the introduction of a PEGylated side chain linked to a sugar moiety in P1, did not change drastically the affinity of the ligand for ADAM17 (IC_50_ = 5.5 nM) with respect to the parent compound. The glycoconjugate compound **2** maintained also the selectivity for ADAM17 over the other tested enzymes, typical of the parent compound JG26.

Then, the antiviral properties of the three ADAM17 inhibitors were assayed on SARS-CoV-2-infected human lung adenocarcinoma Calu-3 cells after cytotoxicity evaluation with the aim to prove their efficacy to impair the alternative entry pathway of SARS-CoV-2 in host cells.

**Fig. 3:**
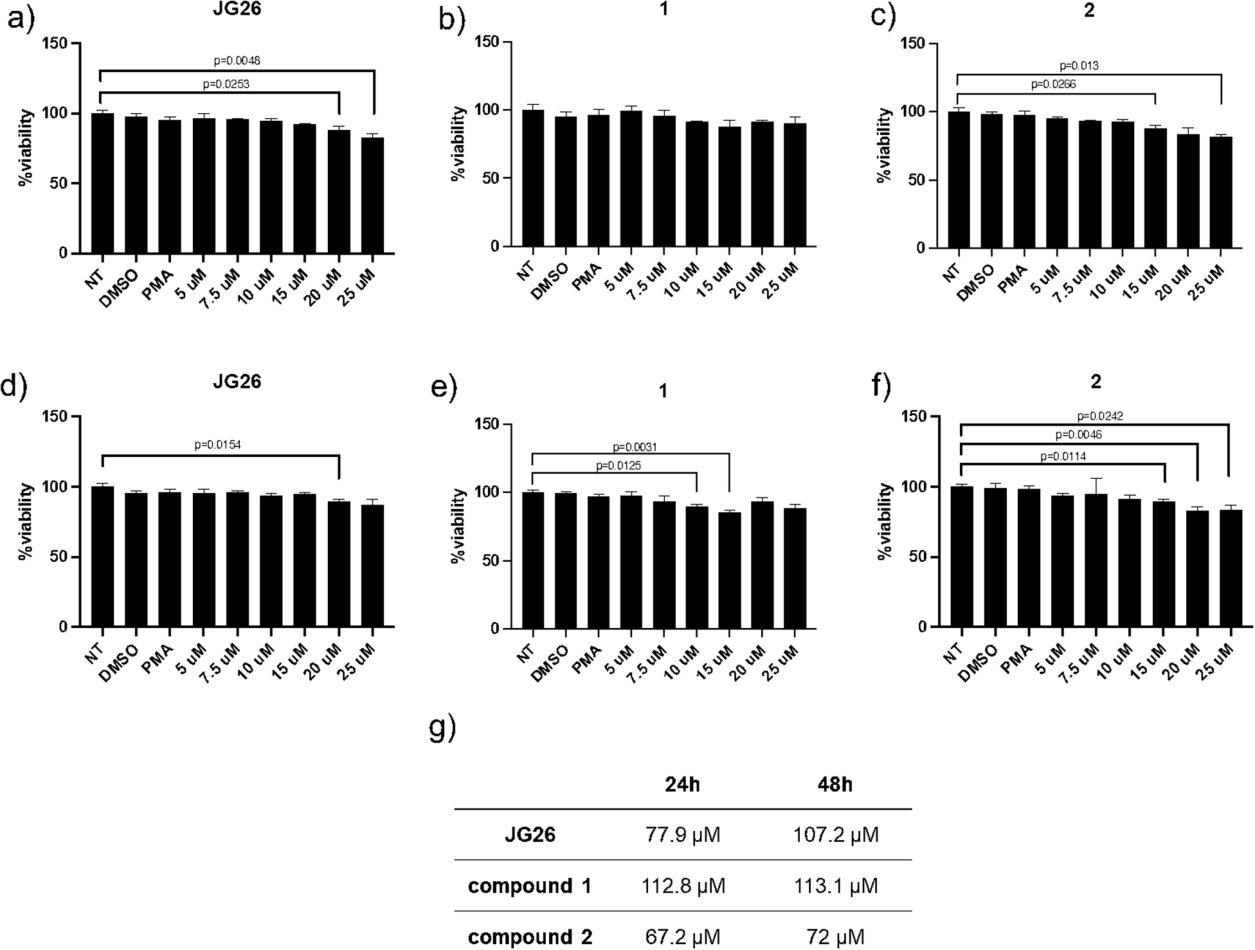
Cytotoxicity in Calu-3 cells evaluated by MTT assay. Cells were cultured in the presence of different concentrations of compound JG26, compound **1** and compound **2** for 24 (panels **a**-**c**) and 48 h (panels **d**-**f**). (**g**) CC_50_ (cytotoxic concentration 50%, µM) values reported for each compound. Data were analyzed by one-way ANOVA followed by Dunnett’s T3 multiple comparison post hoc test. The data are presented as mean ± SD (*n* = 3). NT, not treated; PMA, phorbol 12-myristate 13-acetate

All the cytotoxicity data resulted to be normally distributed by Kolmogorov-Smirnov test. Despite we reported some differences in the distribution of variance considering both 24 h and 48 h of treatment (see supplementary Table S1 for one-way ANOVA statistics), as shown in Fig. 3 none of the treatments significantly affected cell viability in the concentration range analyzed, at both 24 (Fig. 3a – c, all % viability > 80%) and 48 h (Fig. 3d – f, all % viability > 80%) of treatment, as confirmed by CC_50_ evaluation, which showed 50% cell death for concentration higher than 25 µM (Fig. 3g).

**Fig. 4:**
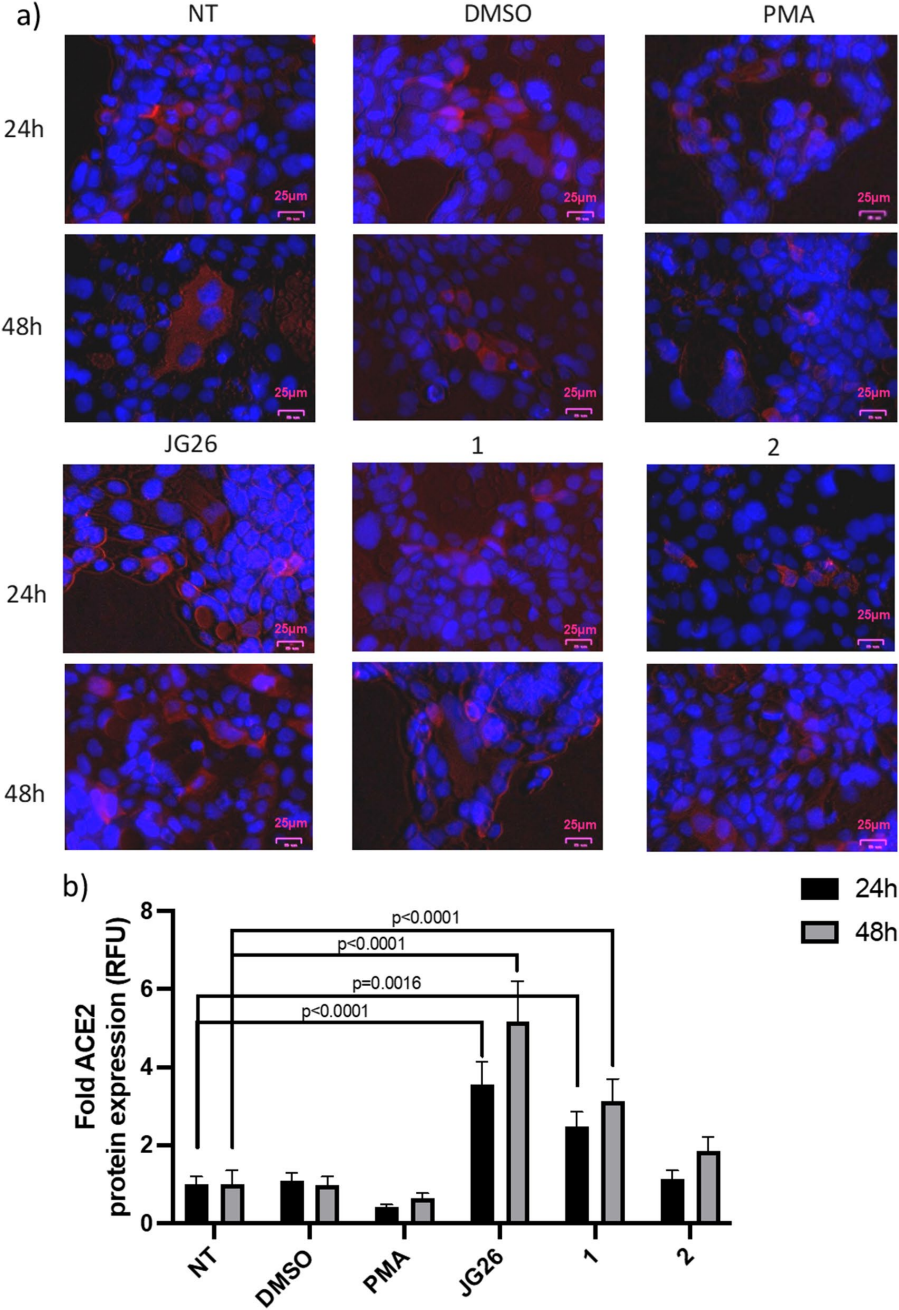
ACE2 expression in SARS-CoV-2-infected Calu-3 cells after treatment with the tested compounds (25 µM) for 24 and 48 h using immunofluorescence staining. (a) Representative images of immunofluorescence for ACE2 (red); nuclei were stained with DAPI (blue). Images were taken with microscope Nikon Eclipse TE2000S, scale bars (low right) denote 25 μm. (b) Immunofluorescence levels of ACE2 expressed in RFU, quantified by QuPath software. Data were analyzed by two-way ANOVA followed by Dunnett’s multiple comparison post hoc test. The data are presented as mean ± SD (*n* = 3). NT, not treated; ACE2, angiotensin-converting enzyme 2; PMA, phorbol 12-myristate 13-acetate; RFU: relative fluorescence units

Based on these results, we selected the highest concentration tested of 25 µM for further experiments. Considering the role of ACE2 receptor in viral entry into host cells [30, 31], and the ability of ADAM17 to shed it from cell surface [32], we analyzed ACE2 expression on Calu-3 cell surface by immunofluorescence after 24 and 48 h of treatment with the three compounds, at the concentration of 25 µM (Fig. 4 and supplementary Figure S1). As expected, the treatment with the ADAM17 inductor PMA slightly decreased ACE2 expression, compared to untreated samples, while DMSO did not show any difference compared to untreated control (Fig. 4).

We reported an increased ACE2 expression on cell membranes after treatment with the three compounds tested, which was more evident after treatment with JG26 and compound **1** (Fig. 4a, b, F_5,29_ = 47.84, *p* < 0.0001, two-way ANOVA ), at both 24 h and 48 h of treatment (Fig. 4a, b, F_1,29_ = 8.935, *p* = 0.0057, two-way ANOVA). This result suggests that the effect of these two compounds is strengthened by prolonged treatments, even if the main effect seems related to the treatment itself.

It should be considered that, even if a higher ACE2 expression might increase SARS-CoV-2 cell entry, a decreased ACE2 shedding may affect the ability of SARS-CoV-2 to infect new cells, particularly those characterized by low ACE2 expression [15]. Moreover, it has been described that ADAM17 inhibitors can decrease SARS-CoV-2 lung cell infection in a TMPRSS2 protease-independent manner [10].

This hypothesis is also supported by evidence that treatment with PMA, inducer of ADAM17, is associated with increased SARS-CoV-2 infection [10], which may involve an increased ACE2 shedding.

**Fig. 5:**
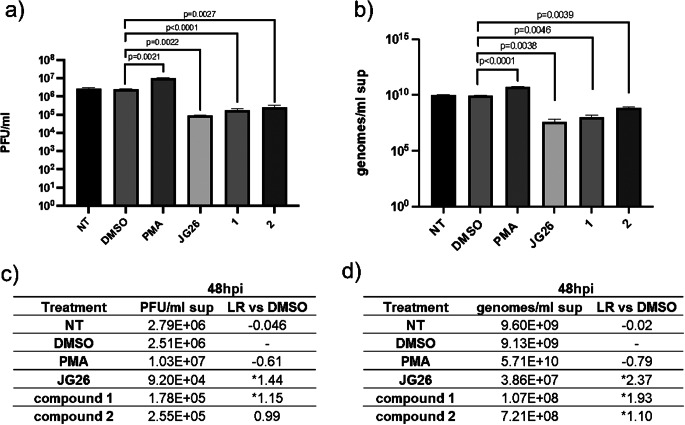
The antiviral activity of JG26, compound **1** and compound **2** on Calu-3 SARS-CoV-2-infected cell. (**a**) Quantification by plaque assay of SARS-CoV-2 infective particles in supernatants of treated cells 48 h post-infection. (**b**) Quantification by RT-qPCR of SARS-CoV-2 genomes in supernatants of treated cells 48 h post-infection. (**c**, **d**) Data of viral load logarithmic reduction (LR) in compounds-treated Calu-3 cells in comparison to DMSO-treated (vehicle) cells, infected with SARS-CoV-2, calculated with the results of plaque assay (**c**) or RT-qPCR (**d**). Data were analyzed by one-way ANOVA followed by Dunnett’s T3 multiple comparison post hoc test. The data are presented as mean ± SD (*n* = 3). NT, not treated; PMA, phorbol 12-myristate 13-acetate; PFU, plaque-forming unit; hpi, hours post-infection. *Logarithmic Reduction greater than 1 log, corresponding to a > 90% viral load decrease

Our data suggested a treatment with compounds at 25 µM concentration for 48 h as the most effective on ACE2 maintenance on cell surface, thus this condition was selected to perform the following assays. All the compounds were tested for their antiviral activity on infected Calu-3 cells and the results were compared to the untreated infected sample. The antiviral activity was tested by both plaque assay (Fig. 5a) and genome titration by real-time PCR on infected Calu-3 supernatants (Fig. 5b). Data were also reported as Log Reduction (LR) and considered relevant when LR > 1, corresponding to a 90% reduction of viral load.

As expected, PMA treatment induced a sensible SARS-CoV-2 infection progression [10], while no significant difference was observed after treatment with DMSO, showing LR < 1 (Fig. 5c, d). On the contrary, the treatment with all the three compounds analyzed showed a significant reduction in SARS-CoV-2 infection (Fig. 5a, b, F_5,12_ =274.2, *p* = 0.0016 and F_5,12_ = 811.9, *p* = 0.0002, respectively, one-way ANOVA), which was more evident in the presence of JG26 treatment, as reported by the higher LR, compared to the vehicle (Fig. 5c, d, LR = 1.44 and 2.37 in plaque assay and genome titration, respectively). On the contrary, a lower concentration of JG26 (10 µM) did not show any effect on viral infection compared to vehicle-treated cells (supplementary Figure S2).

On the basis of these results, EC_50_ and SI have been defined for the three tested compounds, confirming JG26 as the most effective and safest compound to be used for a future in vivo treatment for SARS-CoV-2 infection (Table 2).

**Table 2:** EC_50_ (µM) and SI evaluation for compound JG26 and its derivatives (compound **1**, compound **2)**

Compound	EC_50_ (µM)	SI
**JG26**	8.1	13.23
compound **1**	12.7	8.91
compound **2**	13.6	5.29

EC_50_, half maximal effective concentration; SI: selectivity index calculated as CC_50_/EC_50_ ratio, using CC_50_ values reported in Fig. 3 after 48 h of treatment; CC_50_, cytotoxic concentration 50%

The main effect on SARS-CoV-2 infection observed in the presence of JG26 treatment is in line with the reduced ACE2 shedding reported, since this could affect the ability of the virus to enter in new cells characterized by no or low amount of the receptor.

**Fig. 6:**
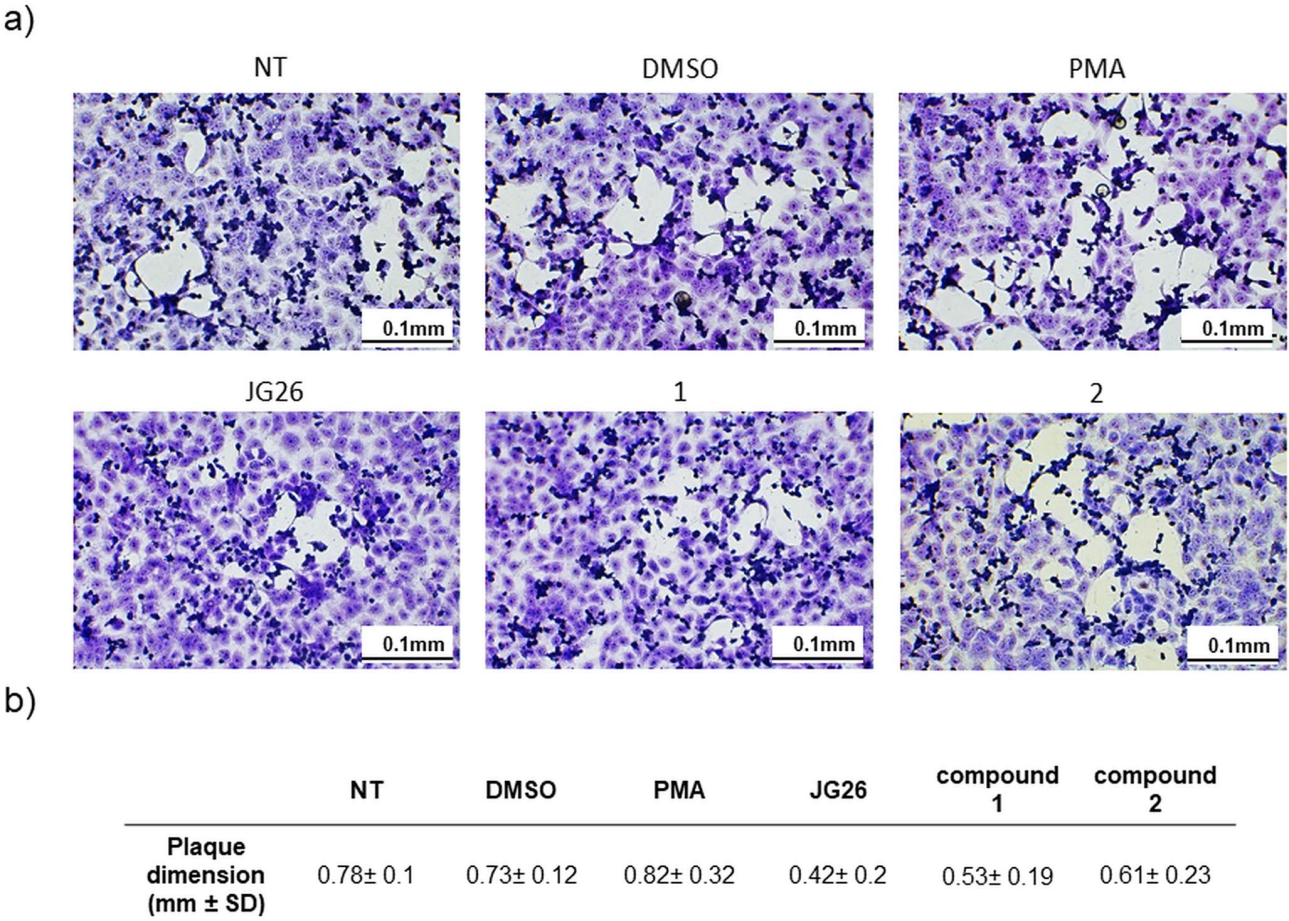
The impact of the treatment with compound JG26 and its derivatives (compound **1**, compound **2**) on SARS-CoV-2 plaque morphology. (**a**) Representative images of SARS-CoV-2 plaques on VERO E6 cells stained with crystal violet 0.1%. Images were taken with NeXcope NE620 microscope, scale bar (low right) 0.1 mm. (**b**) The impact of the treatment on plaque diameter calculated as mean value of 10 plaques for each condition. Diameter sizes were measured using NeXcope visualization software (Capture V.2.0). Data were analyzed by one-way ANOVA followed by Dunnett’s T3 multiple comparison post hoc test. NT, not treated; PMA, phorbol 12-myristate 13-acetate

Finally, the observation by microscopy of the cytopathic effect revealed a differential dimension of SARS-CoV-2 plaques formed in Vero E6 cells after infection with supernatants derived from the Calu-3 experiment. Compared to untreated cells, we reported a trend in the reduction of plaque dimension in the presence of treatment with all the three compounds tested (Fig. 6a, b, and supplementary Figure S3, F_5,54_ = 1.544, *p* = 0.3132, one-way ANOVA) that was not observed considering both PMA and DMSO treatment, despite it was not statistically significative.

These results are consistent with the reduced SARS-CoV-2 infection rate and suggest an effect on viral replication performance and spread, possibly due to ACE2 reduced shedding.

## Discussion

This study investigated the compound JG26 and its dimeric (compound **1**) and glycoconjugate (compound **2**) derivatives for their potential antiviral effects against SARS-CoV-2 infection, using an in vitro system based on Calu-3 human lung cells. These molecules are synthetic inhibitors of ADAM17, which is involved in the release of ACE2, a key receptor for viral entry [33–35]. We found that the selective ADAM17 inhibitor JG26 partially inhibited both ACE2 receptor shedding and SARS-CoV-2 infection at 25 µM concentration.

The observed differences between IC_50_ values on isolated enzymes and those on cells are not uncommon in drug development and can be attributed to several factors like enzyme accessibility and protein binding in a cellular environment. These inconsistencies highlight the importance of using multiple models to evaluate drug efficacy and underscore the complexity of translating in vitro findings to cellular and in vivo contexts. The results showed no cytotoxic effects for any of the three compounds tested at concentrations up to 25 µM after 48 h of treatment (Fig. 3). Specifically, JG26 and compound **1** were found to effectively reduce ADAM17-mediated ACE2 shedding in Calu-3 cells (Fig. 4). This finding was confirmed by antiviral activity assays, which demonstrated JG26 as the most effective compound in controlling viral infection at 25 µM concentration, as evidenced by both viral genome titration (Fig. 5) and plaque formation assays (Fig. 6). JG26 antiviral activity was evident at 25 µM but not at 10 µM, and this result can be attributed to its concentration-dependent efficacy, which is influenced by factors such as binding affinity, mechanism of action, cellular uptake, stability, and assay conditions, which need to be further investigated. JG26 was already found active at a concentration of 30 µM in reducing CD16 shedding in PBMC co-cultured with hepatitis C virus-infected HuH7.5 cells [36] and these previous findings supported our results. Since the low efficacy of compound **2** could not be explained based on the affinity for ADAM17, further studies will be necessary to rationalize this finding, based for example on the reduction of ADAM17 expression by specific siRNA or blocking antibodies. These more specific assays would allow to exclude the possibility that non-specific effects of these inhibitors are causing the decrease in SARS-CoV-2 replication. Moreover, experiments on infected Calu-3 cells testing a combination of JG26 with inhibitors of TMPRSS2, such as camostat, in comparison with JG26 alone could demonstrate a synergy of effects on blocking the SARS-CoV-2 entry into host cells with important translational implications.

This study provides important initial insights but presents also some limitations: cell lines are simplified models that do not fully replicate the complexity of human tissues and organs, lacking the complex cell-to-cell interactions and microenvironmental factors present in vivo. Further studies will allow to prove the anti-inflammatory effect of JG26 on infected Calu-3 cells or on K18-hACE2 mice infected with SARS-CoV-2.

In conclusion, we have identified JG26 as a promising ADAM17 inhibitor for managing SARS-CoV-2 infection by hampering the viral entry process, targeting a host cell protein rather than viral proteins like the papain-like protease (PLPro), main protease (Mpro/3CLpro), and RNA-dependent RNA polymerase (RdRp), extensively investigated in early studies [37, 38].

Additionally, ADAM17 contributes to the development of inflammatory conditions associated with COVID-19 [16]. In 2022 Hedges et al. showed that an anti-human ADAM17 mAb tested in SARS-CoV-2 infected transgenic mice led to a reduction of inflammatory damage but also to a higher viral burden in mice lungs [39]. This in vivo study supported an antiviral role of ADAM17. In contrast, other recent studies employing lung cell culture models showed opposite results [10]. Despite differing opinions on the significance of this protease in SARS-CoV-2 infection, ADAM17-mediated shedding of ACE2 may lead to increased levels of sACE2, potentially enhancing viral spread in tissues with low or absent ACE2 expression [13]. The role of sACE2 in augmenting viral infectivity has been a topic of interest in recent studies, reporting that sACE2 can promote infection by forming a complex with SARS-CoV-2 S protein and vasopressin which facilitates virus entry into host cells [15, 40]. Consequently, the inhibition of ADAM17 could have a dual therapeutic effect: a modulation of the cytokine storm associated with COVID-19 and an attenuation of SARS-CoV-2 infection.

Given the diverse roles of ADAM17 in the development of COVID-19, this strategy highlights the potential of ADAM17 inhibitors as a promising option for treating COVID-19, emphasizing the need for additional research to assess their effectiveness and safety in clinical settings.

## Electronic supplementary material

Below is the link to the electronic supplementary material.

Supplementary Material 1

## Data Availability

No datasets were generated or analysed during the current study.

## Abbreviations

ACE2: Angiotensin-converting enzyme 2
ADAM17: A Disintegrin And Metalloproteinase 17
ADAMTS: A disintegrin and metalloproteinase with thrombospondin type I repeats
APMA: *p*-Aminophenylmercuric acetate
CC_50_: Cytotoxicity concentration 50%
COVID-19: Coronavirus disease 2019
DIPEA: N, N-diisopropiletilammina
EC_50_: Half maximal effective concentration
GAG: Glycosaminoglycan
GlcNAc: β-*N*-acetyl-D-glucosamine
IC_50_: Half maximal inhibitory concentration
MMP: Matrix metalloproteinase
MTT: 3-(4,5-Dimethylthiazol-2-yl)-2,5 diphenyl tetrazolium bromide
PMA: Phorbol 12-myristate 13-acetate
sALCAM: Soluble activated leukocyte cell adhesion molecule
SARS-CoV-2: Severe acute respiratory syndrome coronavirus 2
SD: Standard deviation
SI: Selectivity index
TACE: Tumor necrosis factor-α‐converting enzyme
TFA: Trifluoroacetic acid
TMPRSS2: Transmembrane protease serine 2
