# Supplementary material for: JG26 attenuates ADAM17 metalloproteinase-mediated ACE2 receptor processing and SARS-CoV-2 infection in vitro
Source: Pharmacol Rep. 2024 Sep 18;77(1):260–73. doi: 10.1007/s43440-024-00650-0 (PMC11743353; doi:10.1007/s43440-024-00650-0)
Supplement: Supplementary file 1 — Supplementary Material 1 [file 43440_2024_650_MOESM1_ESM.docx]

**SUPPORTING INFORMATION FOR**

JG26 attenuates ADAM17 metalloproteinase-mediated ACE2 receptor processing and SARS-CoV-2 infection in vitro

*Valentina Gentili ^1§^, Silvia Beltrami^1§^, Doretta Cuffaro^2^, Giorgia Cianci^1^, Gloria Maini^1^, Roberta Rizzo^1,3^, Marco Macchia^2^, Armando Rossello ^2^, Daria Bortolotti^1‡^* and Elisa Nuti ^2‡^**

^1^ Department of Chemical, Pharmaceutical and Agricultural Sciences, University of Ferrara, Ferrara, Italy

^2^ Department of Pharmacy, University of Pisa, Pisa, Italy

^3^ LTTA, Clinical Research Center, University of Ferrara, Ferrara, Italy

* Corresponding authors: Daria Bortolotti, Department of Chemical, Pharmaceutical and Agricultural Sciences, University of Ferrara, Via Luigi Borsari 46, 44121, Ferrara, Italy email: brtdra@unife.it, Tel.+39 0523455398; Elisa Nuti, Department of Pharmacy, University of Pisa, Via Bonanno 6, 56126 Pisa, Italy, email: elisa.nuti@unipi.it, Tel. +39 050 2219551

*^§^* V.G. and S.B. contributed equally to this work.

*^‡^* E.N. and D.B. contributed equally to this work.

**Table of Contents**

**I. Representative NMR spectra of final compounds (1, 2 and JG26) S2**

**II. Table S1 S5**

**III. Figure S1 ……..…………………………………..………………………… S6**

**Figure S2…………………………………………………………………………………………... S7**

**Figure S3 ……..…………………..…………………………………………… S8**

1. **Representative NMR spectra of final compounds (1, 2 and JG26)**

Compound **1:** ^1^H NMR (400 MHz, CD_3_OD)

**
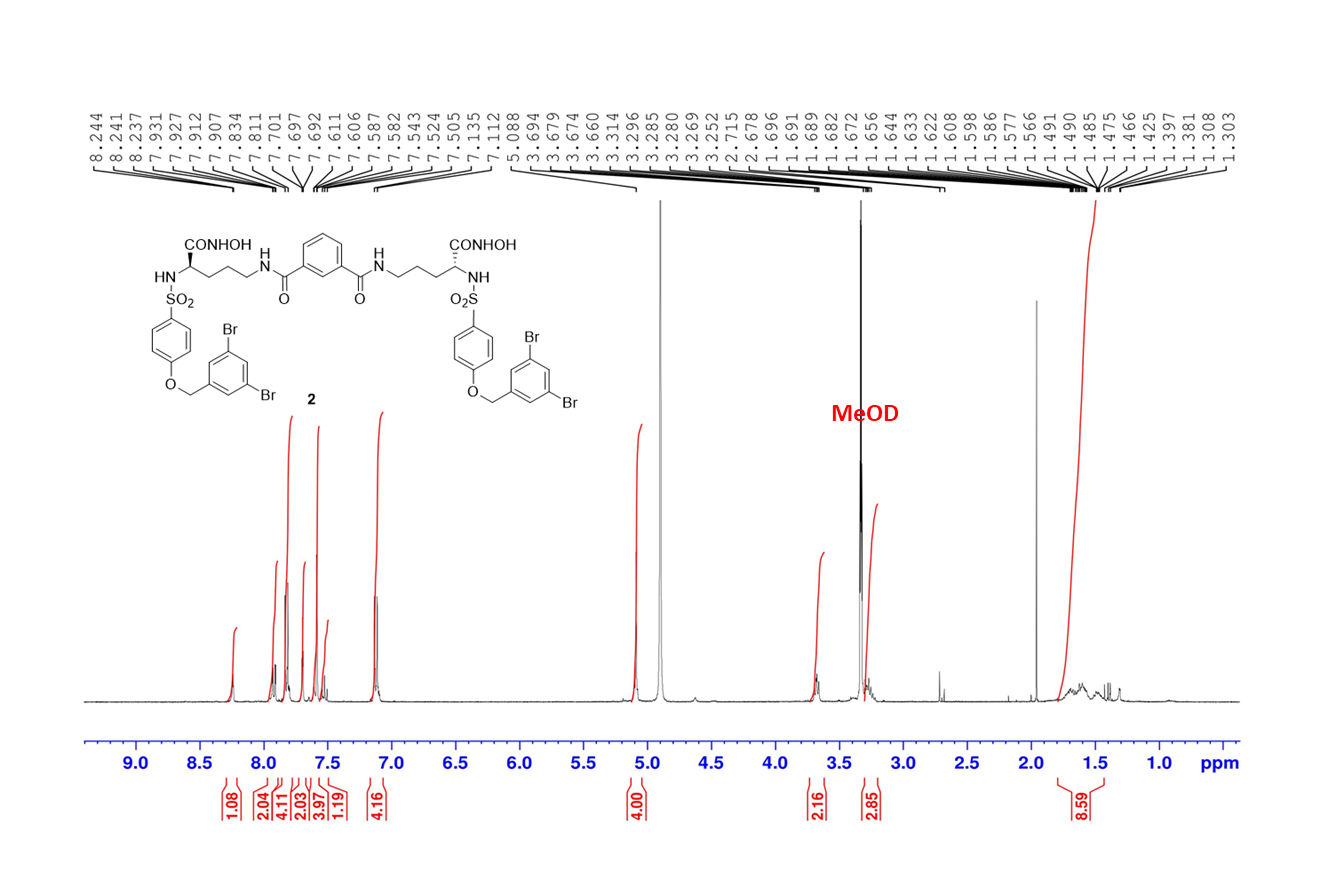
**

1

Compound **1**: ^13^C NMR (100 MHz, CD_3_OD)

**
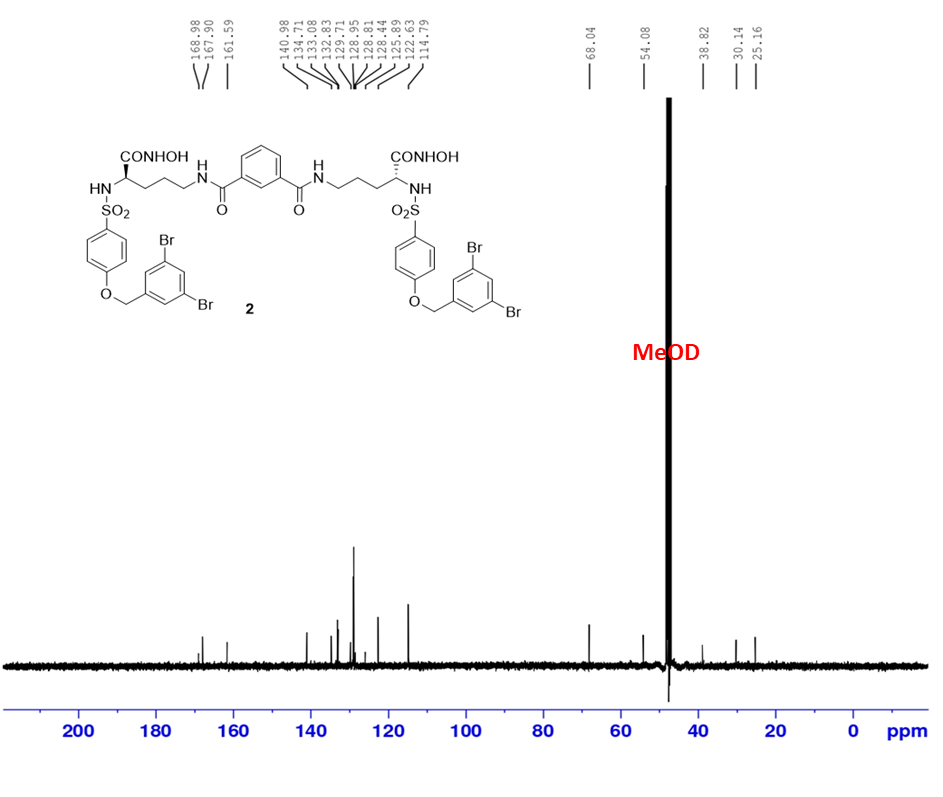
**

1

Compound **2:** ^1^H NMR (400 MHz, DMSO)

Compound **2**: ^13^C NMR (100 MHz, MeOD)

Compound **2:** ^1^H-^1^H COSY bidimensional analysis

Compound **JG26:** ^1^H NMR (400 MHz, DMSO)

Compound **JG26**: ^13^C NMR (100 MHz, DMSO)


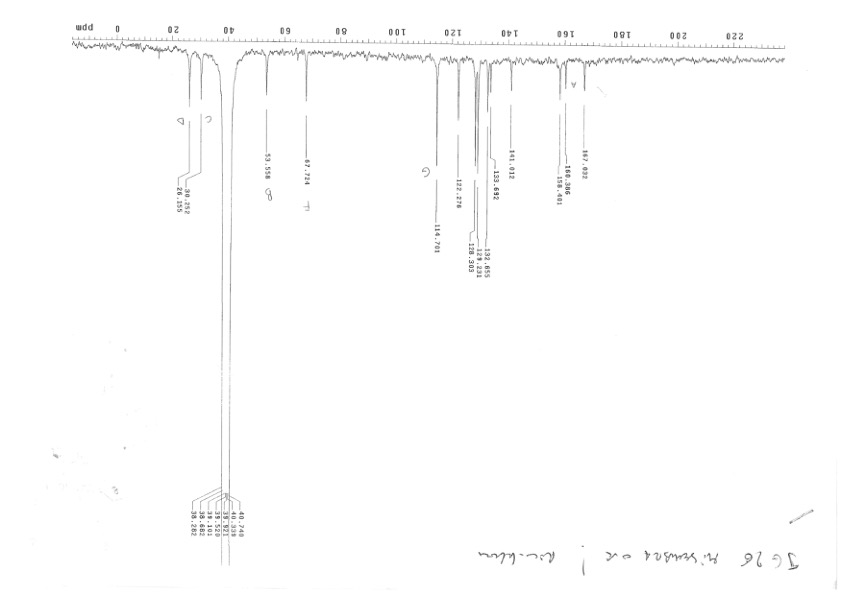


1. Supplementary Table 1.


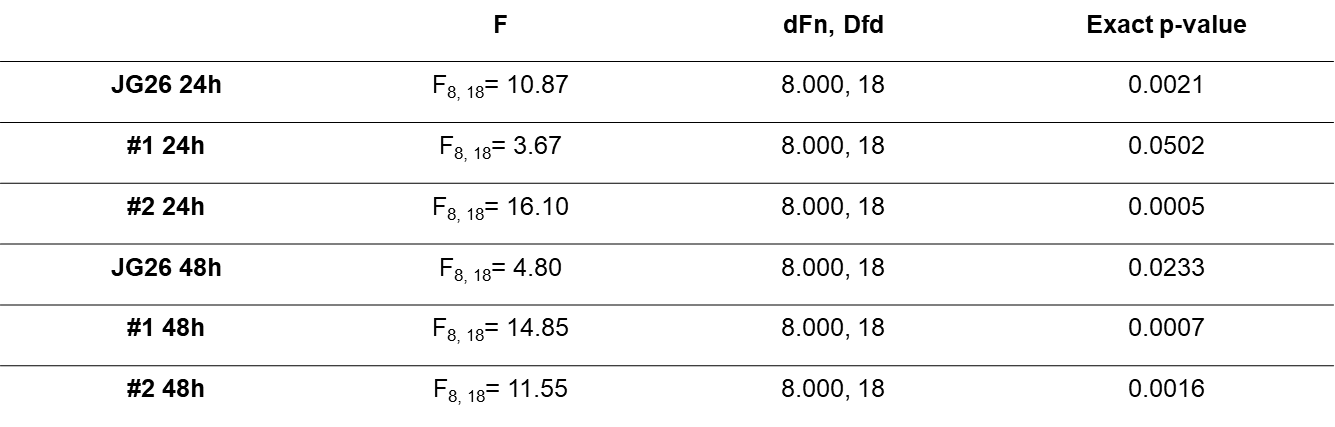


Table S1: One-way ANOVA test analysis. For each condition values for F, dF (DFn and DFd) and exact p-value are reported.

1. **Supplementary Figures**


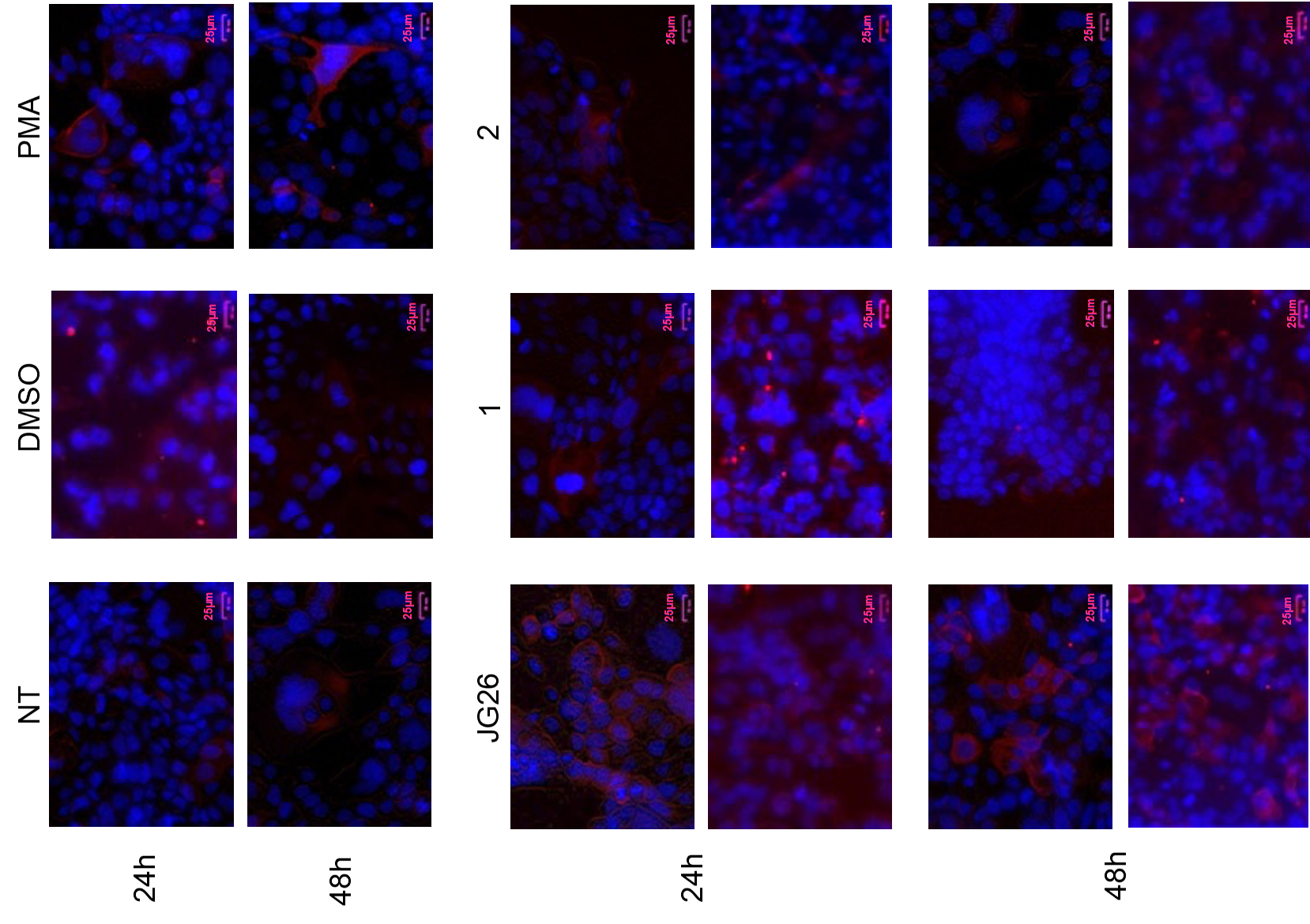


Figure S1: ACE2 expression evaluation in SARS-CoV-2-infected Calu-3 cells after treatment with 25 μM compound for 24 and 48 h by immunofluorescence. Images were taken with microscope Nikon Eclipse TE2000S, magnification bar (low right) 25 µm.


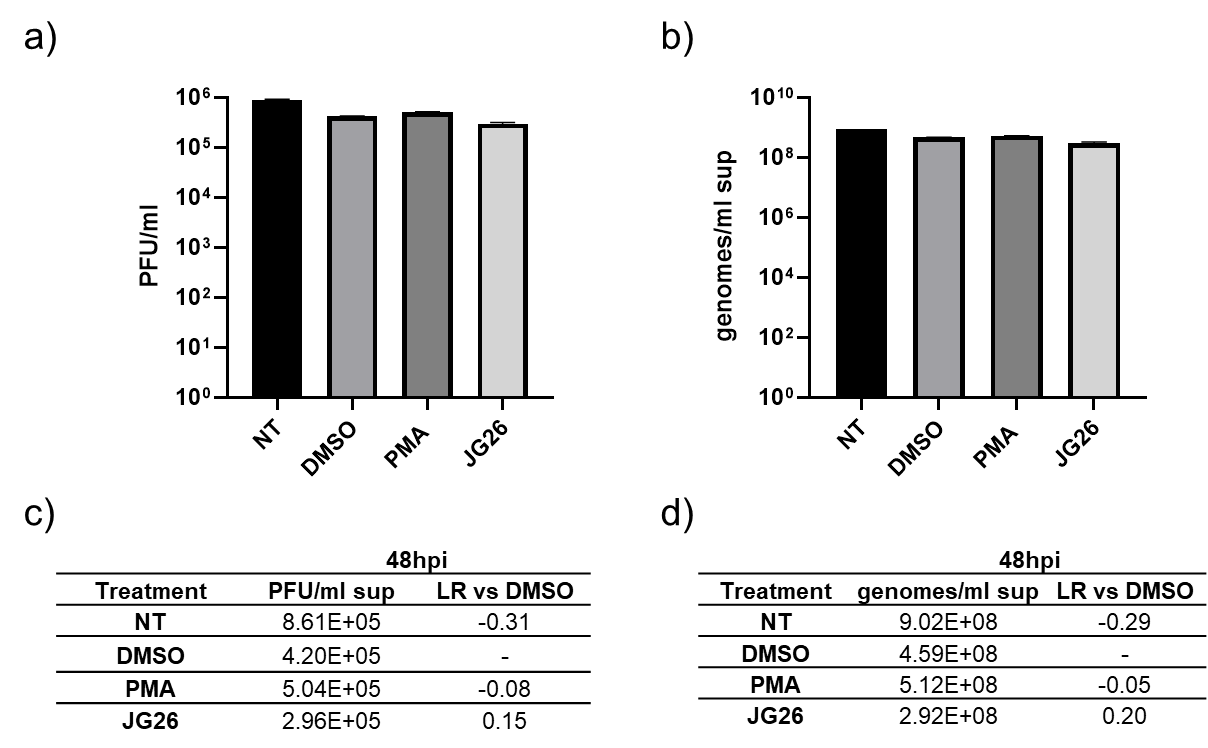


**Figure S2**: Evaluation of the antiviral activity of JG26 at 10µM concentration on Calu-3 SARS-CoV-2-infected cell supernatants by the plaque assay and RT-qPCR (a,b). Data were reported also as viral load log reduction (LR) in comparison to Calu-3 DMSO (vehicle) infected with SARS-CoV-2 (c and d). Experiments were run in triplicates and reported as mean ± SD. Data were analyzed by one-way ANOVA followed by Dunnett’s T3 multiple comparison post hoc test. NT, not treated; PMA, phorbol 12-myristate 13-acetate; PFU, plaque-forming unit; hpi, hours postinfection.


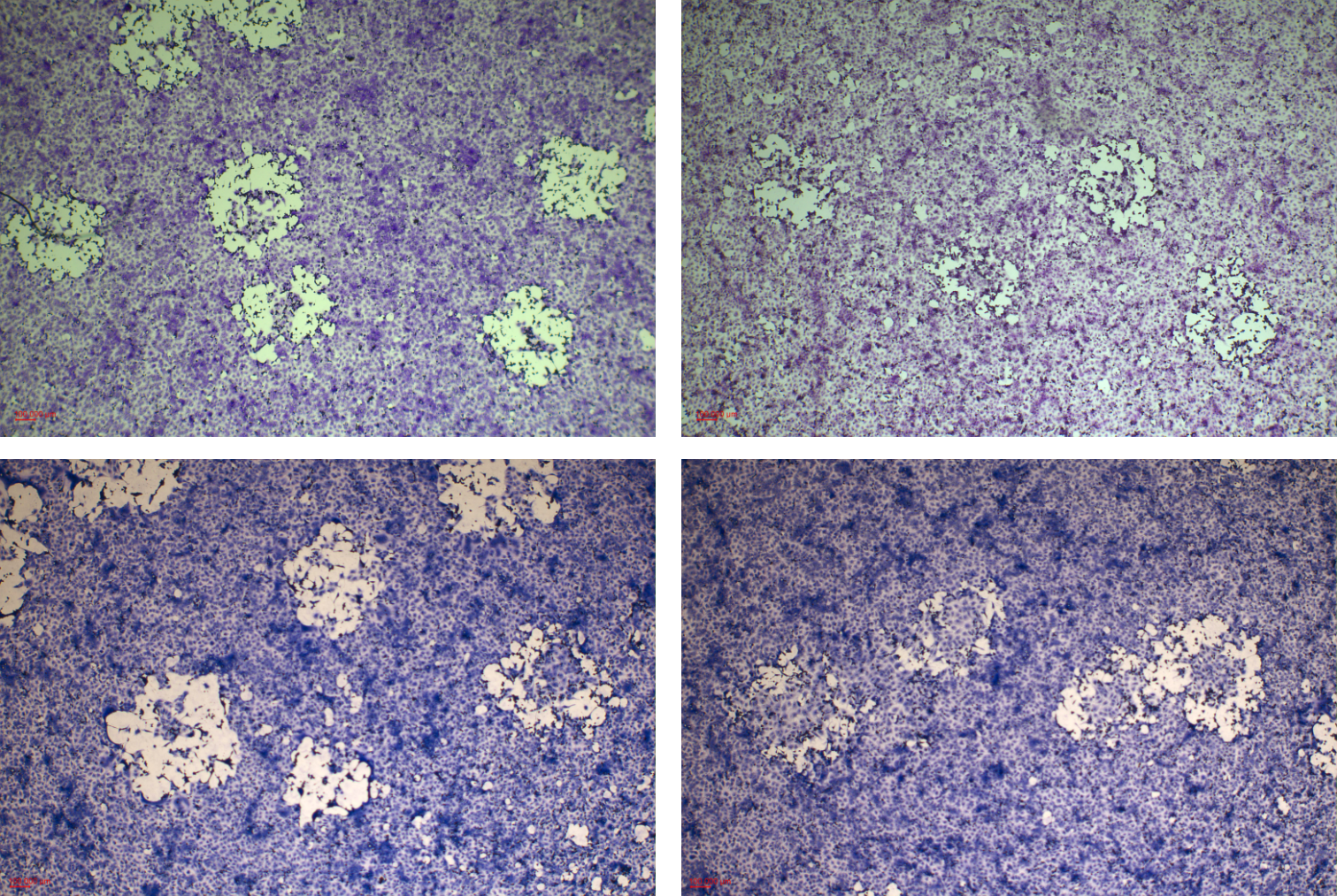


Figure S3: Representative images of not treated plaques (on the left) and JG26 derived plaques (on the right). Images were taken with microscope NeXcope NE620, 4x magnification, scale bar: 0.1 mm.
